# Gastrointestinal Bacterial and Methanogenic Archaea Diversity Dynamics Associated with Condensed Tannin-Containing Pine Bark Diet in Goats Using 16S rDNA Amplicon Pyrosequencing

**DOI:** 10.1155/2014/141909

**Published:** 2014-01-02

**Authors:** Byeng R. Min, Sandra Solaiman, Raymon Shange, Jong-Su Eun

**Affiliations:** ^1^Department of Agricultural and Environmental Sciences, Tuskegee University, Tuskegee, AL, USA; ^2^Department of Animal, Dairy, and Veterinary Sciences, Utah State University, Logan, UT, USA

## Abstract

Eighteen Kiko-cross meat goats (*n* = 6) were used to collect gastrointestinal (GI) bacteria and methanogenic archaea for diversity measures when fed condensed tannin-containing pine bark (PB). Three dietary treatments were tested: control diet (0% PB and 30% wheat straw (WS); 0.17% condensed tannins (CT) dry matter (DM)); 15% PB and 15% WS (1.6% CT DM), and 30% PB and 0% WS (3.2% CT DM). A 16S rDNA bacterial tag-encoded FLX amplicon pyrosequencing technique was used to characterize and elucidate changes in GI bacteria and methanogenic archaea diversity among the diets. *Proteobacteria* was the most dominant phylum in goats with mean relative abundance values ranging from 39.7 (30% PB) to 46.5% (control) and 47.1% (15% PB). Other phyla individually accounted for fewer than 25% of the relative abundance observed. Predominant methanogens were *Methanobrevibacter* (75, 72, and 49%), *Methanosphaera* (3.3, 2.3, and 3.4%), and *Methanobacteriaceae* (1.2, 0.6, and 0.7%) population in control, 15, and 30% PB, respectively. Among methanogens, *Methanobrevibacter* was linearly decreased (*P* = 0.05) with increasing PB supplementation. These results indicate that feeding PB selectively altered bacteria and methanogenic archaeal populations in the GI tract of goats.

## 1. Introduction

Studies on gastrointestinal (GI) microorganisms have traditionally depended on the use of anaerobic cultivation techniques [1] which only can detect an estimated 11% of the total bacterial populations present in the rumen [2]. Molecular methodologies developed over the past decade now enable researchers to examine the diversity of the gut microflora independent of cultural methods. Bacterial diversities within the GI tract of humans [3, 4] and rumen of beef cattle [5, 6] have been investigated in recent years as a result of development of 16S rDNA-based analysis, yet similar data on the microbiomes of bacteria and methanogenic archaea diversity in the lower GI tract of meat goats are limited. In addition, there is no clear information on the prevalence of methanogenic and microbial populations in the hindgut of animals other than human and swine.

Plant tannins (condensed (CT) and hydrolysable tannins) are polyphenolic compounds of relatively high molecular weight with the capacity to form complexes mainly with proteins due to the presence of a large number of phenolic hydroxyl groups [7]. They are ubiquitously spread in nutritionally important forage trees, shrubs, legumes, cereals, and tree barks. The effects of tannins on ruminant production have been extensively published in the past, and among them beneficial effects of tannins on animal production system have been much focused, with particular interest in their positive effects on protein metabolism, prevention of frothy bloat, and modification on rumen microbial population [7]. Microbial population changes in the gut when animals were fed CT-containing diets were reported using a 16S PCR technique in sheep [8] and rat [9]. In addition, the use of rapid sequencing technologies combined with molecular methods is becoming a prevalent standard for evaluating the microbiome of animals [10]. However, there is still a lack of information on the microbial diversity in the gut of ruminants such as sheep and goats. To date, little research has been focused on changes in microbial population diversity, particularly methanogenic archaeal population, and no study has measured the fecal microbial population in response to feeding diets containing different dietary concentrations of CT-containing pine bark (PB). In our previous study, addition of PB in goat diets improved growth performance, reduced fecal egg counts, and favorably modified ruminal fermentation [11]. In the present study, therefore, we utilized a bacterial tag-encoded FLX-titanium amplicon pyrosequencing (bTEFAP) method that is able to perform diversity analyses of fecal microbial populations. We evaluated the fecal microbiomes from 18 Kiko-cross meat goats fed PB containing diets. Hence, our principal objective of this study was to measure the effects of CT-containing PB supplementation as a feed supplement on GI methanogenic archaea and bacterial diversity dynamics in meat goats.

## 2. Materials and Methods 

Care and handling of all experimental animals were conducted under protocols approved by the Tuskegee University Institutional Animal Care and Use Committee.

### 2.1. Experimental Animals and Diets

Eighteen Kiko crossbred male goat kids (*Capra hircus*; initial body weight (BW) = 31.0 ± 1.49 kg) were stratified by BW and randomly assigned to one of 3 experimental treatment groups (*n* = 6). Goats were individually housed indoors in pens of approximately 1.2 m^2^ with elevated floors and were fed grain mixes with different amounts (0, 15, and 30% dry matter (DM) PB) of PB and long bermudagrass hay (BGH; *Cynodon dactylon*) at 85 : 15 on an as-fed basis. An adjustment period of 4 weeks allowed goats to become acclimated to pen living and routine feeding and to allot time for proper diet adjustment prior to study initiation. In wk 1, all animals were fed a diet without PB (control diet). Starting on wk 2, in the adjustment period, experimental diets were gradually fed to animals in a stepwise increasing fashion, and at the end of wk 4, all animals were fed whole, preassigned experimental diets (Table 1).

Experimental diets (Table 1) contained different amounts of the CT-containing ground PB replacing ground wheat straw (WS; *Triticum aestivum*). Experimental treatments included the control diet (0% PB), 15% PB, and 30% PB on an as-fed basis. The WS contained negligible CT (0.03%), but concentrations of neutral detergent fiber (NDF) and nonfiber carbohydrates were similar between PB and WS [11]. The fresh PB was donated by a wood-processing company (West Fraser Timber Co. Ltd., Opelika, AL) and air-dried under the shed before processing. Freshly dried PB (10.3% CT) was ground (Hammer Mill Model 1250; Lorenz MFG Co., Benson, MN) to approximately 3 mm particle. The WS (0.03% CT) was also ground (3 mm) and incorporated in the grain mix portion of the diets. Concentration of CT in 15% PB and 30% PB diets was 1.63% and 3.20% DM, respectively (Table 1). The inclusion rate of PB was chosen, as we previously reported that beneficial effects of CT in the diet on sheep performance may occur in the range of 2 to 4% CT of diet DM [7, 11]. Mixtures containing ground PB and WS were commercially prepared at the local feed mill (Eclectic Feed Mill, Eclectic, AL). Experimental diets met requirements of experimental animals for growth and BW gain according to the NRC [12]. Diets were isonitrogenous and isocaloric but differed in NDF, lignin, and CT concentrations; lignin and CT concentrations were greater in 15% and 30% PB rations, whereas NDF concentration was decreased compared with control diet.

### 2.2. Sample Collection and Laboratory Analysis

After completion of adaptation period for 4 wk, total fecal collection was performed for 7 d to all goats. Animals were fed once a day at 8:00 h and had free access to water and a trace mineral salt block. Grain mixes and hay were offered separately, and refusals were recorded daily. Amounts of feed offered were adjusted every day to maintain the preferred daily refusal rate of 5 to 10%.

During the collection periods, total individual fecal sample from the metabolism crate was collected at 9:00 am (24 h overnight collection) during 7 d with two different experimental periods. Feed and orts samples for individual animals were collected daily during the collection period, dried at 60°C for 48 h, ground to pass a 1 mm screen (standard model 4; Arthur H. Thomas Co., Swedesboro, NJ), and stored for subsequent analyses. Daily portions of ground samples were composited for each animal and analyzed for DM, crude protein (CP), acid detergent lignin, ether extract, and ash according to the methods described by AOAC [13]. Nitrogen for diet sample was determined using a Kjeldahl N, and CP was calculated by multiplying N by 6.25. The NDF and ADF concentrations were sequentially determined using an ANKOM^200/220^ Fiber Analyzer (ANKOM Technology, Macedon, NY). Sodium sulfate heat stable amylases (Sigma Aldrich Co., St. Louis, Mo) were used in the procedure for NDF determination and pretreatment with heat stable amylase (Type XI-A from *Bacillus subtilis*; Sigma-Aldrich Corporation, St. Louis, MO). Acetone (70%) extractable CT in grain mixes were determined using a butanol-HCL colorimetric procedure [7, 11].

### 2.3. DNA Extraction

Genomic bacterial DNA was isolated from 0.3 g of fecal samples according to the method described in the Power soil DNA mini Kit (Power Soil, West Carlsbad, CA). Extracted DNA (2 *μ*L) was quantified using a Nanodrop ND-1000 spectrophotometer (Nyxor Biotech, Paris, France) and run on 0.8% agarose gel with 0.5 M tris-borate-EDTA (TBE) buffer. The samples were then transported to the Research and Testing Laboratory (Lubbock, TX) for PCR optimization and pyrosequencing analysis. Bacterial tag-encoded FLX amplicon pyrosequencing PCR was carried out according to procedure described previously [10, 14].

### 2.4. bTEFAP Sequencing PCR

The bTEFAP and data processing were performed as described previously [10]. All DNA samples were adjusted to 100 ng/*μ*L. A 100 ng (1 *μ*L) aliquot of each sample's DNA was used for a 50 *μ*L PCR reaction. The 16S universal eubacterial primers 530F (5′-GTG CCA GCM GCN GCG G) and 1100R (5′-GGG TTN CGN TCG TTG) were used for amplifying the 600 bp region of 16S rRNA genes. HotStar Taq Plus Master Mix Kit (Qiagen, Valencia, CA) was used for PCR under the following conditions: 94°C for 3 min followed by 32 cycles of 94°C for 30 sec; 60°C for 40 sec and 72°C for 1 min; and a final elongation step at 72°C for 5 min. A secondary PCR was performed for FLX (Roche, Nutley, NJ) amplicon sequencing under the same condition by using designed special fusion primers with different tag sequences as: LinkerA-Tags-530F and LinkerB-1100R [10]. The use of a secondary PCR prevents amplification of any potential bias that might be caused by inclusion of tag and linkers during initial template amplification reactions. After secondary PCR, all amplicon products from different samples were mixed in equal volumes and purified using Agencourt Ampure beads (Agencourt Bioscience Corporation, MA).

### 2.5. bTEFAP FLX Massively Parallel Pyrosequencing

In preparation for FLX sequencing, the DNA fragments size and concentration were accurately measured by using DNA chips under a Bio-Rad Experion Automated Electrophoresis Station (Bio-Rad Laboratories, CA) and a TBS-380 Fluorometer (Turner Biosystems, CA). A sample of double-stranded DNA containing 9.6 × 106 molecules/*μ*L of average size 625 bp was combined with 9.6 million DNA capture beads and then amplified by emulsion PCR. After bead recovery and bead enrichment, the bead-attached DNA was denatured with NaOH, and sequencing primers were annealed. A two-region 454 sequencing run was performed on a 70 × 75 GS PicoTiterPlate (PTP) by using a Genome Sequencer FLX System (Roche, Nutley, NJ). It should be noted that 100 total samples were run within this same FLX 2-region sequencing reaction. The additional 79 tagged samples were associated with unrelated studies. All FLX related procedures were performed according to manufacturer's instructions (Genome Sequencer FLX System). A custom script written in manufacture's instruction was utilized to generate all possible combinations of 10-mer oligonucleotide tags with GC % between 40 and 60%. From this pool 20 individual tags were chosen (Table 2).

The resultant individual sample after parsing the tags into individual FASTA files was assembled using CAP3. The ace files generated by CAP3 were then processed to generate a secondary FASTA file containing the tentative consensus (TC) sequences of the assembly along with the number of reads integrated into each consensus. The TC was required to have at least 3-fold coverage. The resulting TC FASTA for each sample was then evaluated using BLASTn [15] against a custom database derived from the RDP-II database [16] and GenBank website at http://www.ncbi.nlm.nih.gov/. The sequences contained within the curated 16S database were both >1200 bp and considered as high quality based upon RDP-II standards.

### 2.6. Data Processing and Statistical Analysis

**Figure 1:**
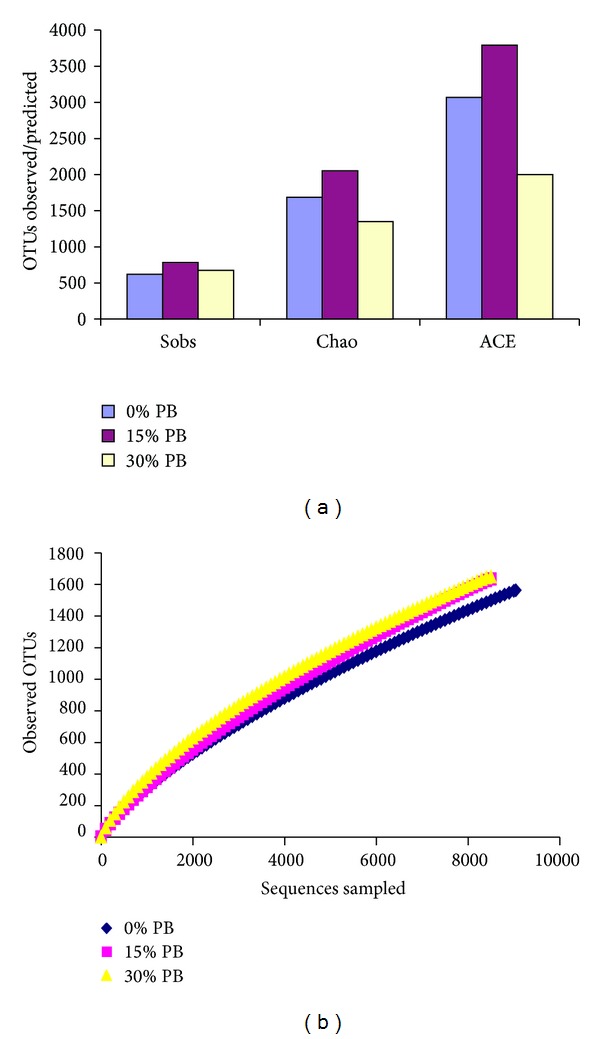
Bacterial richness/diversity estimator (a) and rarefaction curves (b) presented as calculated by MOTHUR at a level of 3% dissimilarity. 0% PB (control), 15% PB, and 30% PB on an as-fed basis.

**Figure 2:**
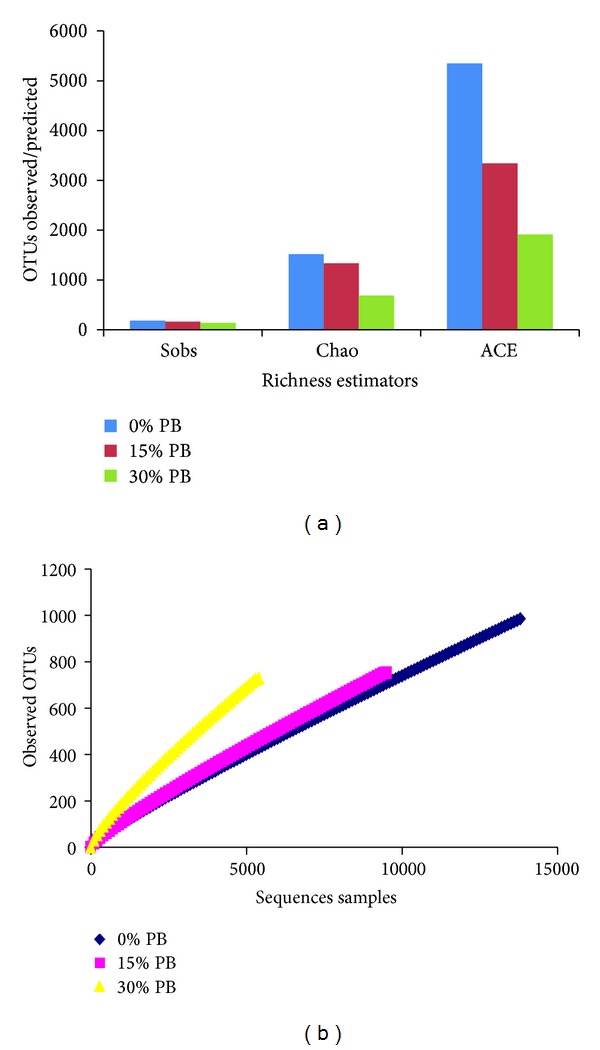
Fecal archaea richness/diversity estimator (a) and rarefaction curves (b) presented as calculated by MOTHUR at a level of 3% dissimilarity. 0% PB (control), 15% PB, and 30% PB on an as-fed basis.

**Figure 3:**
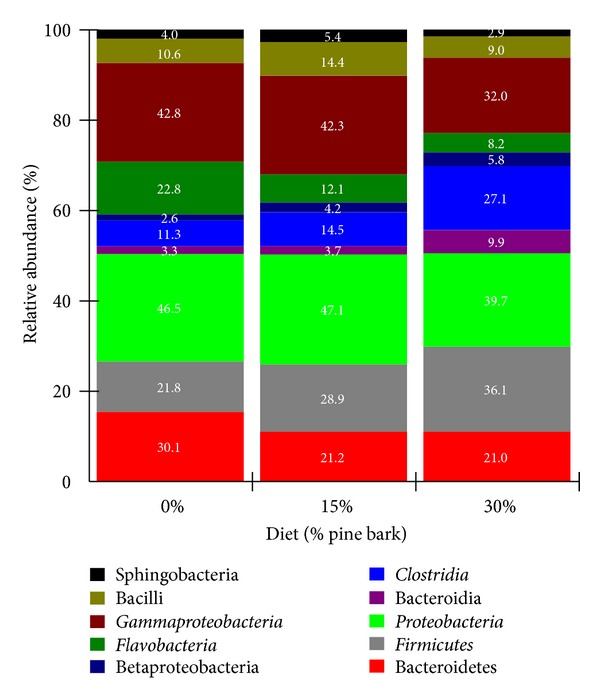
Relative abundance of major taxonomic groups across pine bark (PB) dietary supplementation in growing meat goats. Phyla included in this figure had relative abundance values consistently greater than 1%, as well as the abundant classes of the phylum Proteobacteria. Values presented are the mean percentage. 0% = 0% PB (control), 15% = 15% PB, and 30% = 30% PB on an as-fed basis.

**Figure 4:**
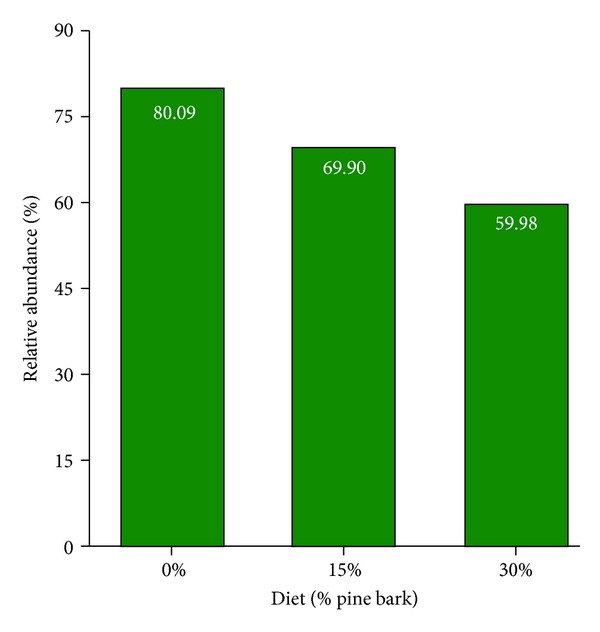
Relative abundance of major taxonomic group (*Euryarchaeota*) across pine bark (PB) dietary supplementation in growing meat goats. Phyla included in this figure had relative abundance values consistently greater than 1%. Values presented are the mean percent. 0% = 0% PB (control), 15% = 15% PB, and 30% = 30% PB on an as-fed basis.

**Figure 5:**
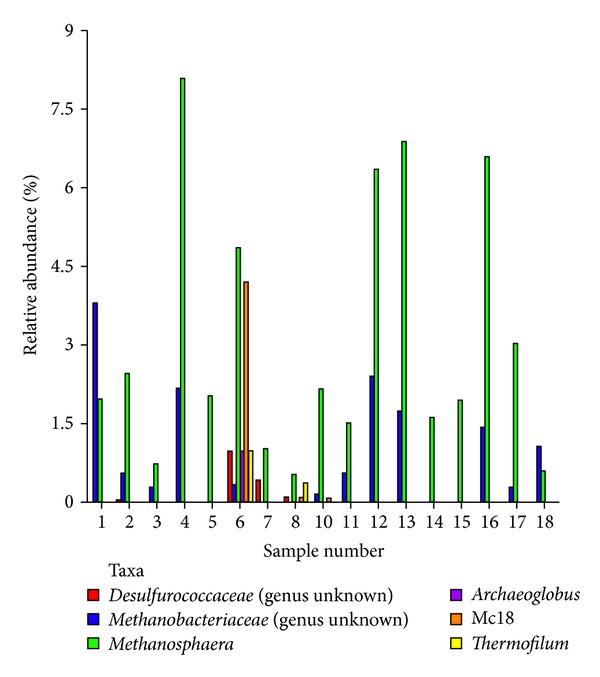
The 6 most abundant archaea genera (*Methanobrevibacter*, *Desulfurococcaceae*,* Methanosphaera*, *Archaeoglobus*, Mc18, and *Thermofilum*) in the hindgut of various levels of pine bark (PB) diets from a common cohort of 17 meat goats. Clustering in the *Y* direction is indicative of abundance, not phylogenetic similarity. 0% PB (control) = sample (or tag) number 1, 2, 3, 4, 5, and 6; 15% PB = tag number 7, 8, 10, 11, and 12; 30% PB = tag number 13, 14, 15, 16, 17, and 18.

**Figure 6:**
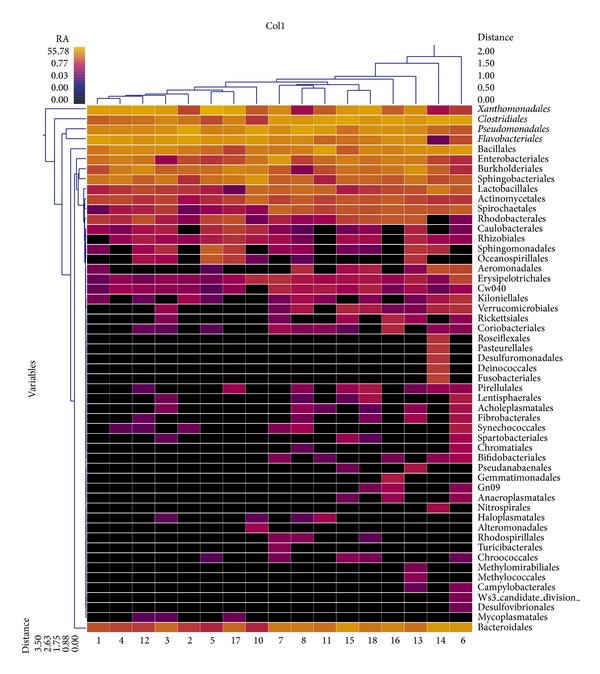
Thermal double dendrogram of the 53 most abundant bacterial genera in the hindgut of various levels of pine bark (PB) diets from a common cohort of 17 meat goats. Clustering in the *Y* direction is indicative of abundance, not phylogenetic similarity. RA = relative abundance; 0% PB (control) = tag number 1, 2, 3, 4, 5, and 6; 15% PB = tag number 7, 8, 10, 11, and 12; 30% PB = tag number 13, 14, 15, 16, 17, and 18.

All statistical analyses (Figures 1 to 6) were performed using the SPSS package (SPSS Inc., v 17.0, Chicago, IL). Relative abundance data are presented as percentages/proportions, but prior to subjection to GLM, they were transformed using the arcsine function for normal distribution prior to analysis. Package of NCSS (NCSS, 2007, v 7.1.2, Kaysville, UT) was used for cluster analysis through which double dendrograms were generated through use of the Manhattan distance method with no scaling and the unweighted pair technique. Raw sequences were submitted to the NCBI Sequence Read Archive (SRA). In addition, quantification of major hindgut bacterial and archaea populations (Tables 3 and 4) was analyzed by the GLM procedure of the SAS (SAS Inst., Cary, NC) in a completely randomized design with the factors examined being three levels of PB supplementation in the diets. Linear and quadratic effects were determined utilizing orthogonal polynomial contrasts for equally spaced treatments. Animals were the experimental unit and treated as a random effect. There were no treatment × period interactions (*P* > 0.10), hence only the main effects are reported for major microbial populations in the result section. Results are reported as least square means.

Quality trimmed sequences were provided with the sequencing services by the Research and Testing Laboratory (Lubbock, TX) [16]. Tags which did not have 100% homology to the original sample tag designation were not included in data analysis. Sequences which were less than 250 bp after quality trimming were not also considered. The B2C2 software [17], which is described and freely available from the Research and Testing Laboratory, was used to deplete samples of definite chimeras. Further processing and operational taxonomic units (OTUs) based analyses were then carried out using the MOTHUR v.1.19.4 [18] suite of algorithms for sequence processing and diversity analysis, including commands for identifying/consolidating unique sequences, filtering, multiple sequence alignment, generating distance matrices, and clustering of sequences into OTU. The resulting clusters were assessed at 3% and 5% dissimilarity to provide the data needed for diversity analysis. Based upon rarefaction [19], it is expected that 0% dissimilarity in sequences provides dramatic overestimation of the species present in a sample. The resulting sequences were then evaluated using the classify.seqs algorithm (Bayesian method) in MOTHUR against a database derived from the Greengenes set using a bootstrap cutoff of 65%. The sequences contained within the curated 16S database were those considered as high quality based upon Greengenes [20] standards and which had complete taxonomic information within their annotations. Clusters at 3% and 5% were then utilized to generate rarefaction curves and the diversity indices ACE [21, 22] as well as unweighted and weighted UniFrac for Principle Coordinate Analysis (PCOA) plots and a Venn diagram.

## 3. Results and Discussion

Despite several decades of studies demonstrating the role of the ruminal and GI microbial diversity in ruminants associated with bacteria and archaea populations, the response of the microbial consortium to feeding various CT-containing diets remains largely unknown. The principal objective of this study was to assess the effects of CT-containing PB supplementation as a feed replacement on gastrointestinal methanogenic archaea and bacterial diversity dynamics in meat goats. The most significant findings in the present study was a decreased predominant fecal methanogenic genera *Methanobrevibacter *population (comprising up to 75% of the archaea population) as the level of PB supplement increased in the diet. The bacterial distribution showed that proteobacteria was the most dominant phyla with mean relative abundance values ranging from 46.5% in control to 47.1% in 15% PB and to 39.7% in 30% PB diets.

### 3.1. Diet Composition

Ingredients and chemical composition of experimental diet, PB, WS, and BGH are presented in Table 1. Goats were provided diets that met all animals' requirements for growth and gain according to NRC [12]. Total CT concentration in the PB and WS was 10.3 and 0.03% DM, respectively. However, grain mixes contained 0.19, 1.63, and 3.20% CT DM for the 0, 15, and 30% PB diet, respectively. All the experimental treatments provided similar nutrient profiles, except CT and acid detergent lignin that were higher in 15 and 30% PB ration. In our previous study, addition of PB in goat diets improved ADG and favorably modified ruminal fermentation [11].

### 3.2. Richness and Diversity Estimates

In the richness and diversity data in bacteria and archaea was presented in Figures 1 and 2, respectively. For bacteria, the maximum OTUs observed across the study were detected in control group, although the highest mean of bacterial richness was detected in the treatment group fed the 15% PB diet (782.6; Figure 1(a)). Maximum OTUs were predicted for CHAO and ACE estimates with similar trends for the same sample as well. The trend of impact of PB in diet (15% > 30% > 0%) was found in richness observations (sobs) that showed that the highest concentration of PB in the goat diet resulted in the lowest amount of bacteria detected in the feces while the control and 15% diet showed very little difference. When calculating richness with Chao and ACE, the distinction between the treatments is more explicit as the 30% diet remained the lowest, followed by the control and then the 15% diet. In calculating rarefaction curves richness estimates was found to be within Figure 1(b). It was hypothesized that bacterial community richness under CT-containing PB supplementation would differ due to inhibition of bacterial growth [23] and reduce fiber digestibility in the rumen [24]. Smith et al. [9] reported that fecal bacterial population was affected by the presence of CT in the rat gastrointestinal tract.

When considering richness observations and estimates for archaeal community in the study, results demonstrate a vastly different trend (Figures 2(a) and 2(b)). Mean values for observed richness were consistently higher in the control treatment for all of the richness calculators. The trend observed of OTUs the highest mean value for the control (181), followed by the 15% diet (164), while the 30% diet remained the lowest (135; Figure 2(a)). The same trend was observed in the Chao and ACE estimates, as well as the OTUs observed in rarefaction. Furthermore, the Chao estimate appears to show the greatest coverage of the rarefaction peak values. The microbial community in the 30% PB supplemented group consistently showed the lowest values for richness in bacteria (Figures 1(a) and 1(b)) and archaea (Figures 2(a) and 2(b)).

It has been reported that the PB contained high level of CT [11] and polyphenolic compounds [25]. This chemical disturbance creates a microbial community with distinct abiotic and biotic characteristics, which impacts the growth and stability of gastrointestinal microbial community [26].

### 3.3. Relative Abundance of Bacterial and Archaea Phyla

Bacterial (Figure 3) and archaeal (Figure 4) community compositions of the feces were examined at descending levels of biological classification to were to determine the effect of PB supplementation on community membership. Detailed phylogenic analyses grouped the fecal bacteria associated bacterial sequences into 20 phyla (including unknown). The relative abundances of the 10 most abundant phyla are presented in Figure 3. The bacterial distribution showed that *Proteobacteria *was the most dominant phylum with mean relative abundance values ranging from 46.5% in control to 47.1 in 15% PB and to 39.7% in 30% PB diets. They include a wide variety of pathogens, such as *Escherichia*, *Salmonella*, *Vibrio*, *Helicobacter*, and many other notable genera [27]. Others are free-living, and include many of the bacteria responsible for nitrogen fixation. Other dominant phyla were *Gammaproteobacteria *(representing 32.0–42.8% of the bacterial sequences in the samples) and *Firmicutes *and *Bacteroidetes* (which represented 21.0 to 36.1% of the bacterial sequences in each sample). The remaining phyla accounted for fewer than 25% of the relative abundance observed and were designated as minor. Of these groups, *Gammaproteobacteria* (*P* < 0.05), *Flavobacteria *(*P* < 0.01), *Proteobacteria* (*P* < 0.05), and *Bacteroidetes* (*P* < 0.05) were linearly decreased (*P* < 0.05) with increasing PB diets. However, *Clostridia* (*P* < 0.01) and *Firmicutes* (*P* < 0.05) were linearly increased with increasing PB supplementation. This has been confirmed by the findings that *Firmicutes*, *Bacteroidetes*, *Actinobacteria*, and *Proteobacteria* were reported to be dominant bacterial phyla in the human gut [28].

The trend with respect to the one dominant archaea community group can be seen in Figure 4, in which the class of *Euryarchaeota* population was linearly decreased  (*P* < 0.01) with increasing PB diets. Collectively, the observed changes in frequency of occurrence depict dramatic shifts in fecal microbial communities associated with changes in diet in goats. It has been classified that *Euryarchaeota*, one of the meanwhile four kingdoms (or phyla) of the archaeal domain, consists of the strict anaerobic methanogens, the extreme halophiles, the hyperthermophilic *Archaeoglobales *and *Thermococcales*, and the cell wall free *Thermoplasmatales *[29, 30].

Tannins are phenolic plant secondary compounds and are widely distributed through the plant kingdom which affect animal performance and gut microorganisms. Tannins exist primarily in CT and hydrolysable tannins [7]. The hydrolysable tannins occur mainly in fruit pods and plant galls and unlike CT their degradation products are absorbed from the small intestine of animals [7] and are potentially toxic to ruminants. In contrast, CT, unlike most compounds, is only partially absorbed in the small intestine in ruminants [31]. Most of the dietary CT reach the colon where simple phenolics and phenols (e.g., phenylacetic, mono- and dihydroxyphenylacetic acids, and protocatecuic acid) are produced by breakdown due to microbial activity [32]. Ultimately, these simple phenolics can be further metabolized to nonaromatics, such as short-chain fatty acids, lactate, succinate, ethanol, and the gases (CO_2_ and H_2_) by the colonic microorganisms [28]. Therefore, the bacterial characterization of the gut microorganisms is surely important, but ultimately the enzymatic activity of its microorganisms becomes vital to map functional metabolic reactions and describe the interaction between host and its microbe to understand metabolism [28].

### 3.4. Diversity and Abundance of Fecal Bacteria and Archaea

For ease of presentation and interpretation, we present prevalent bacterial genera (Figure 5) and archaeal genera (Figure 6) community based on a cutoff value of 0.9% of relative community abundance for inclusion in a double dendogram cluster analysis of individual animal microbial diversity within and among diets in Figures 5 and 6. Overall, animals clustered relatively well within diet and animals. However, 15% PB diet of one (tag number 12) animal clustered more closed to the control diet (0% PB). We attribute “misclassification” of this animal to greater relative abundance of *Xanthomonadales*, *Clostridiales*, *Pseudomonadales*, and *Flavobacteriales* and lower abundance (%) of *Mycoplasmatales *and *Bacteroidales *compared with animals who received PB diet. Similar diversity trends in archaeal populations were observed on the same diets. For archaea, microbial archaea clustered vary between diets, but we classified these animals to greater relative abundance of *Methanobacteriales *and lower abundance of *Archaeoglobus* and *Thermofilum *between diets.

### 3.5. Quantitative Analyses

So far, only a limited number of bacterial species have been identified as being involved in the metabolism of CT and polyphenols, and little is known about the microbial populations in the hindgut of the goat. The metabolic activities of the bacteria and archaea in the gut are very important in determining the healthy functioning and methane gas emissions from the gut. For further quantification of major hindgut bacterial and archaeal species diversity populations in meat goats (*n* = 6) are presented in Tables 3 and 4, respectively. For bacterial species, *Stenotrophomonas koreensis *was the most dominant bacterial species with mean relative abundance values ranging from 23.9% (control) to 9.9% (15% PB) and 17.2% (30% PB). The remaining bacterial species accounted for fewer than 10% of the relative abundance observed. Of these groups, *Flavobacterium gelidilacus *(*P* < 0.02) and *Myroides odoratimimus *(*P* = 0.09) were decreased (*P* < 0.05) with increasing dietary PB concentration. However, *Bacteroides capillosus *(*P* < 0.02), *Clostridium orbiscindens *(*P* < 0.03), and *Oscillospira guilliermondii *(*P* = 0.06) were linearly increased with increasing PB concentration. This finding agrees with the results of a metabolic finger print patterns study of a rat fed CT extract from *Acacia angustissima*. Condensed tannin extracts from *A. angustissima *altered fecal bacterial populations in the gastrointestinal tract, resulting in a shift in the predominant bacteria towards tannin-resistant gram-negative *Enterobacteriaceae *and *Bacteroidetes* [9]. In contrast, the gastrointestinal microbial population was significantly different in cattle, dominated by *Prevotella *(18.2% of total population) in the rumen and *Clostridium *(19.7% of total population) in the feces [29]. Interestingly, the majority of the bacteria in the human gut recorded belongs to the Clostridia group, which is a large component of the gut microbiota [28].

Condensed tannins from green tea catechins, which are monomeric polyphenols, have also been shown to cause a shift in bacteria populations. Polyphenolics from green tea affect gastrointestinal bacteria in humans [28, 30], chickens [33, 34], and pigs [35]. In the human study, 4 weeks of consuming a polyphenolic content equivalent to 10 cups of concentrated green tea were necessary for there to be a great decrease in *Clostridium *species and *Clostridium perfringens*. Other groups of bacteria, including the *Bacteroidaceae*, were not affected [29, 30]. However, Moco et al. [28] reported that diet containing polyphenols (e.g., +catechin) increased the growth of the *Clostridium coccoides*, *Eubacterium rectale*, *Bifidobacterium *spp., and *Escherichia coli*, but significantly decreased the growth of the *Clostridium histolyticum *group. In chickens the levels of cecal lactobacilli increased significantly, while the levels of *Enterobacteriaceae* decreased [33, 34]. All these studies confirmed that CT cause a shift in the bacterial population in the intestinal tract, and a consistent result is that gram-positive bacterial groups are decreased.

The relative abundances of the 4 most abundant (>0.9%) archaea species are presented in Table 4. Predominant hindgut archaeal species among methanogens were *Methanobrevibacter *(75, 72, and 49%), *Methanosphaera *(3.3, 2.3, and 3.4%), and *Methanobacteriaceae *(1.2, 0.6, and 0.7%) population in control, 15, and 30% PB, respectively, and they were linearly decreased or increased with increasing PB concentration (*P* < 0.05). The effect of CT in forages upon interactions between rumen bacteria and plant protein has been proposed [7]. It has been suggested that the proteolysis of soluble proteins in the rumen is affected, primarily by cell-associated proteinases on the rumen bacteria. Furthermore, CT in the diets have been shown to induce changes in morphology of several species of rumen bacteria [7]. Inhibition of rumen microorganisms by CT is probably due to CT:substrate (microorganisms) interactions [7]. One of the possible explanations for the decrease in *Methanobrevibacter *spp. prevalence is assumed to be linked to the substantial relationship between these archaea and protozoa [6] and the protozoa and subsequent reduction in reducing equivalent (H_2_) cross-feeding between archaea and protozoa [36]. Condensed tannins are known to decrease protozoal number [37], and the decrease in methane production could also be mediated through decrease in protozoal number. Tavendale et al. [38] suggested two models of impacting methanogenesis: first, directly affecting activity or population of methanogens, resulting in lower methane emission, and, second, indirectly by reduced hydrogen production by lowering feed degradation. However, the possible mechanisms and effects of CT-containing diets on gastrointestinal methanogenesis and its mode of action are not clearly understood. More *in vivo *studies with a wide range of tannins sources (types) need to be conducted to evaluate the full potential of tannins. In addition, there is a lack of knowledge of a systematic and preventive process control from the anthropogenic emissions of CH_4_, H_2_, and volatile fatty acids and its relationship with microbial community.

## Figures and Tables

**Table 1:** Chemical compositions of pine bark (PB), wheat straw (WS), bermudagrass hay (BGH), and experimental diets fed to Kiko crossbred male goat kids.

Item	Ingredient, %			Experimental diets^1^			SEM
	PB	WS	BGH	Control	15% PB	30% PB	
Ingredient of the grain/PB mix, % as is							
Ground PB	—	—	—	0	15.0	30.0	—
Ground WS	—	—	—	30.0	15.0	0	—
Corn	—	—	—	20.0	20.0	20.0	—
Soybean meal	—	—	—	18.5	20.0	21.0	—
Soy hulls	—	—	—	4.5	5.0	4.0	—
Alfalfa meal	—	—	—	5.0	3.0	3.0	—
Molasses	—	—	—	6.0	6.0	6.0	—
Vitamin and mineral mix	—	—	—	0.5	0.5	0.5	—
Salt	—	—	—	0.5	0.5	0.5	—
NH_4_Cl	—	—	—	0.5	0.5	0.5	—
BGH	—	—	—	15.0	15.0	15.0	—
Chemical composition, % DM (*n* = 3)							
Dry matter	83.6	83.5	91.4	89.7	87.8	87.3	0.77
Crude protein	1.2	4.1	7.3	15.7	16.8	16.1	0.41
Neutral detergent fiber	78.6	79.0	69.2	35.0	31.8	27.5	1.77
Acid detergent fiber	72.1	49.2	37.3	23.7	23.2	23.6	1.42
Ether extract	1.65	0.42	1.51	2.3	2.6	2.5	0.25
Ash	2.3	2.0	4.8	6.4	6.2	5.9	0.31
NFC^2^	17.1	16.7	19.1	42.1	42.5	47.1	1.91
Acid detergent lignin	21.3	8.01	6.3	5.9	9.9	12.4	0.85
Condensed tannins^3^	10.3	0.03	0.04	0.19	1.63	3.20	0.19

^1^Control (0% PB), 15% PB, and 30% PB on an as-fed basis. Except bermudagrass hay, all ingredients were incorporated in the grain mixes.

^2^Nonfiber carbohydrates = 100 – CP – NDF – ether extract – ash.

^3^Condensed tannins (CT) are relative to a purified quebracho CT standard.

SEM: standard error of the mean.

**Table 2:** Primer sequences utilized for goats samples during bTEFAP.

Name	Primer sequence (5′-3′)
454-F30	GCCTCCCTCGCGCCATCAGCGCACTACGTGTGCCAGCMGCNGCGG
454-F31	GCCTCCCTCGCGCCATCAGCGCAGCTGTTGTGCCAGCMGCNGCGG
454-F32	GCCTCCCTCGCGCCATCAGCGCATACAGTGTGCCAGCMGCNGCGG
454-F33	GCCTCCCTCGCGCCATCAGCGCATCTATAGTGCCAGCMGCNGCGG
454-F34	GCCTCCCTCGCGCCATCAGCGCATTGGTGGTGCCAGCMGCNGCGG
454-F35	GCCTCCCTCGCGCCATCAGCGCCAGAAAAGTGCCAGCMGCNGCGG
454-F36	GCCTCCCTCGCGCCATCAGTGTGACGTACGTGCCAGCMGCNGCGG
454-F37	GCCTCCCTCGCGCCATCAGTGTGTGCATAGTGCCAGCMGCNGCGG
454-F38	GCCTCCCTCGCGCCATCAGTGTGTCCTCAGTGCCAGCMGCNGCGG
454-F39	GCCTCCCTCGCGCCATCAGTGTGCATCACGTGCCAGCMGCNGCGG
454-F40	GCCTCCCTCGCGCCATCAGTGTGCCTAGAGTGCCAGCMGCNGCGG
454-F41	GCCTCCCTCGCGCCATCAGTGTACATAGTGTGCCAGCMGCNGCGG
454-F42	GCCTCCCTCGCGCCATCAGTGTACATTGAGTGCCAGCMGCNGCGG
454-F43	GCCTCCCTCGCGCCATCAGTGTACATTGTGTGCCAGCMGCNGCGG
454-F44	GCCTCCCTCGCGCCATCAGTGTACCAACAGTGCCAGCMGCNGCGG
454-F45	GCCTCCCTCGCGCCATCAGTGTACCAACTGTGCCAGCMGCNGCGG
454-F46	GCCTCCCTCGCGCCATCAGTGTACCAATCGTGCCAGCMGCNGCGG
454-F47	GCCTCCCTCGCGCCATCAGTGTACCAGATGTGCCAGCMGCNGCGG
454-F48	GCCTCCCTCGCGCCATCAGTGTACCCATAGTGCCAGCMGCNGCGG
454-F49	GCCTCCCTCGCGCCATCAGTGTACAGGGTGTGCCAGCMGCNGCGG
454-F50	GCCTCCCTCGCGCCATCAGTGTACCTATCGTGCCAGCMGCNGCGG
linkerB-1100R	GCCTTGCCAGCCCGCTCAGGGGTTNCGNTCGTTG

**Table 3:** Quantification of major hindgut bacterial diversity present in meat goat (*n* = 6) as analyzed using bTEFAP pyrosequencing method^1^.

Item	Diets^2 ^			SEM	*P* values^3^	
	Control	15% PB	30% PB		L	Q
*Acinetobacter haemolyticus *	1.1	1.2	0.2	0.06	0.32	0.54
*Acinetobacter lwoffii *	7.0	6.5	0.3	3.70	0.22	0.56
*Acinetobacter radioresistens *	1.4	4.2	1.1	0.92	0.80	0.03
*Acinetobacter rhizosphaerae *	1.1	1.5	0.9	0.47	0.76	0.39
*Acinetobacter schindleri *	3.4	8.5	5.3	0.82	0.46	0.11
*Bacteroides capillosus *	0.5	1.1	2.7	0.60	0.02	0.52
*Clostridium orbiscindens *	0.02	1.4	2.3	0.48	0.03	0.92
*Comamonas aquatica *	2.4	3.9	4.7	1.43	0.25	0.88
*Enterobacter hormaechei *	0.8	3.3	2.0	0.95	0.42	0.16
*Escherichia albertii *	0.6	1.5	0.9	0.53	0.73	0.25
*Escherichia fergusonii *	0.6	1.9	1.2	0.60	0.49	0.18
*Flavobacterium columnare *	0.4	2.0	0.5	0.83	0.91	0.18
*Flavobacterium gelidilacus *	5.81	1.2	1.7	1.14	0.02	1.10
*Flavobacterium succinicans *	1.7	0.2	1.3	1.80	0.58	0.15
*Myroides odoratimimus *	7.8	4.5	1.2	2.58	0.09	0.99
*Oscillospira guilliermondii *	0.99	1.2	3.0	0.69	0.06	0.38
*Sphingobacterium faecium *	1.2	4.4	0.53	1.09	0.83	0.16
*Sphingobacterium mizutaii *	1.8	0.6	1.4	0.41	0.40	0.07
*Stenotrophomonas koreensis *	23.9	9.9	17.2	7.40	0.53	0.28
*Rummeliibacillus pycnus *	8.54	9.9	6.9	0.35	0.75	0.63

^1^Data were presented with a cutoff value of 0.9%.

^2^Control (0% PB), 15% PB, and 30% PB on an as-fed basis.

^3^Polynomial contrasts for equally spaced treatments.

L: linear effect; Q: quadratic effect.

**Table 4:** Quantification of hindgut methanogenic archaea diversity present in meat goat (*n* = 6) as analyzed using bTEFAP pyrosequencing method^1^.

Item	Diets^2^			SEM	*P* values^3^	
	Control	15% PB	30% PB		L	Q
*Methanobrevibacter* spp.	74.8	71.7	49.6	8.84	0.05	0.43
*Methanosphaera* spp.	1.18	0.61	0.74	0.45	0.51	0.57
*Methanobacteriaceae* spp.	3.35	2.31	3.44	1.01	0.95	0.44

^1^Data were presented with a cutoff value of 0.9%.

^2^Control (0% PB), 15% PB, and 30% PB on an as-fed basis.

^3^Polynomial contrasts for equally spaced treatments. L: linear effect; Q: quadratic effect.
